# Effect of Instant Cooked Giant Embryonic Rice on Body Fat Weight and Plasma Lipid Profile in High Fat-Fed Mice

**DOI:** 10.3390/nu6062266

**Published:** 2014-06-13

**Authors:** Soo Im Chung, Tae Hyeong Kim, Catherine W. Rico, Mi Young Kang

**Affiliations:** 1 Department of Food Science and Nutrition, Brain Korea 21 Plus, Kyungpook National University, Daegu 702-701, Korea; E-Mails: zizibe0312@nate.com (S.I.C.); ckwrico@gmail.com (C.W.R.); 2 CJ Cheil Jedang Foods Research and Development, Seoul 152-050, Korea; E-Mail: rice@cj.net

**Keywords:** body fat, giant embryo mutant, instant cooked rice, plasma lipid

## Abstract

The comparative effects of instant cooked rice made from giant embryo mutant or ordinary normal rice on body weight and lipid profile in high fat-fed mice were investigated. The animals were given experimental diets for seven weeks: normal control (NC), high fat (HF), and HF supplemented with instant normal white (HF-NW), normal brown (HF-NB), giant embryonic white (HF-GW), or giant embryonic brown (HF-GB) rice. The HF group showed markedly higher body weight, body fat, plasma and hepatic triglyceride and cholesterol concentrations, and atherogenic index relative to NC group. However, instant rice supplementation counteracted this high fat-induced hyperlipidemia through regulation of lipogenesis and adipokine production. The GB rice exhibited greater hypolipidemic and body fat-lowering effects than the GW or NB rice. These findings illustrate that the giant embryo mutant may be useful as functional biomaterial for the development of instant rice with strong preventive action against high fat diet-induced hyperlipidemia and obesity.

## 1. Introduction

Rice (*Oryza sativa* L.) is one of the most important agricultural crops in the world and the staple food in many Asian countries. While it is mostly consumed as cooked rice, rice-based products, such as rice noodles, breakfast cereals, and rice milk, have been produced to increase its market value. Nowadays, due to modern lifestyles, instant foods are becoming widely popular. Consumers with busy lives and limited time prefer the convenience of instant foods and quick meals that require less preparation time. Various food companies in Asia and North America have developed different types of instant cooked rice, such as whole grain, organic, and instant brown or white rice. Normally produced by precooking the rice, followed by drying to a low moisture level (≤12%), and then packaging [1], instant rice takes about five minutes or less to cook at home.

In recent years, rice scientists and plant breeders have developed rice cultivars with enhanced functional properties, such as high-amylose rice that have high dietary fiber content, beta-carotene-enriched golden rice, giant embryonic rice with high nutritional content, and pigmented rice with strong antioxidant activity [2,3,4]. The giant embryonic rice, in particular, is mutant rice with an enlarged embryo and contains higher amounts of protein, vitamins, essential amino acids, and minerals compared with the normal embryo rice [5]. Moreover, it was reported to have high amounts of γ-oryzanol, tocopherol, and γ-aminobutyric acid (GABA) [2], a neurotransmitter that could control blood pressure and reduce the lipid level in blood [5].

With the rising incidence of obesity and other metabolic disorders across the globe and the growing cost of medical care, there is an increasing demand for natural products and functional foods with body weight-lowering effects and hypolipidemic activity. Although various types of instant cooked rice are already available in the market, food companies are still developing this product with improved nutritional qualities and health-promoting properties using different rice grains. Currently, a new instant cooked rice using a giant embryonic cultivar (Seonong 17) as raw material is being developed in Korea. The present study was conducted to evaluate the effect of instant cooked rice made from a giant embryo mutant, in comparison with that of the instant ordinary normal rice, on the body weight and plasma lipid profile in mice under high fat diet conditions. The enzymes and hormones associated with lipid metabolism were also analyzed.

## 2. Experimental Section

### 2.1. Materials and Chemicals

Four kinds of instant cooked rice (Hatbahn) samples (normal white rice, normal brown rice, giant embryonic white rice, and giant embryonic brown rice) were provided by CJ CheilJedang Corp. (Seoul, Korea). They were freeze-dried and ground into powder using a grinder machine (HMF-3250S, Hanil Electronics, Seoul, Korea). The chemicals such as ethanol, triethanolamine, ketamine-HCl, potassium phosphate buffer, calcium chloride, magnesium chloride, and Triton X-100 were purchased from Merck KGaA (Darnstadt, Germany). All other chemicals used were obtained from Sigma-Aldrich Inc. (Steinhein, Germany).

### 2.2. Analysis of the Proximate and Nutritional Compositions

The proximate compositions of the rice samples were analyzed based on the methods of AOAC [6]. The GABA contents were measured using an amino acid analyzer (L-8800, Hitachi Ltd., Tokyo, Japan) following the method described by Kwak [7]. The γ-oryzanol contents were determined according to the method of Chakuton *et al.* [8]. Analysis of vitamins and minerals was performed using the AOAC method [6]. The resistant starch contents were determined based on a modified Englyst method [9]. The proximate and nutritional compositions of the rice samples are presented in Table 1.

**Table 1:** Proximate and nutritional compositions of the instant cooked rice samples.

Component	Normal White Rice	Normal Brown Rice	Giant Embryonic White Rice	Giant Embryonic Brown Rice
*Proximate composition (% dry basis)*				
Moisture	59.7	57.2	59.9	55.6
Crude protein	2.4	3.3	2.7	3.5
Crude fat	0.2	0.2	0.4	0.5
Ash	0.1	0.7	0.1	0.7
Carbohydrate	37.4	37.3	36.5	38.2
Dietary fiber	0.2	1.3	0.4	1.5
*Nutritional composition (mg/100 g)*				
GABA	2.2	4.3	3.9	5.7
γ-oryzanol	0.8	2.0	2.2	2.9
Vitamin B1	0.1	0.2	0.2	0.3
Vitamin E	0.1	0.5	0.8	0.9
Niacin	0.2	0.3	0.3	0.4
Zinc	1.4	1.5	1.7	1.8
Calcium	8.2	8.5	8.8	9.0
Resistant starch	0.8	1.0	0.8	1.0

### 2.3. Animals and Diets

Forty-eight male C57BL/6N mice (four-week-old), weighing 12 g, were obtained from Orient Inc. (Seoul, Korea). The animals were individually housed in a stainless steel cage in a room maintained at 25 °C with 50% humidity and 12/12 h light/dark cycle. They were fed with pelletized chow diet for two weeks and randomly divided into six dietary groups (*n* = 8). The first and second groups were fed with normal control (NC) and high fat (HF) diets, respectively. The other four groups were fed with HF diet supplemented with instant cooked rice powder: normal white rice (HF-NW), normal brown rice (HF-NB), giant embryonic white rice (HF-GW), and giant embryonic brown rice (HF-GB). The composition of the experimental diet (Table 2) was based on the AIN-76 semisynthetic diet [10]. The mice were fed daily with experimental diets for seven weeks and allowed free access to food and water. Their feed intake and weight gain were measured daily and weekly, respectively. At the end of the experimental period, the mice were anaesthetized with ketamine-HCl following a 12-h fast. Blood samples were drawn from the inferior vena cava into a heparin-coated tube and centrifuged at 1000× *g* for 15 min at 4 °C to obtain the plasma. The body organs (liver, heart, and kidney) and adipose tissues (epididymal, perirenal, and inguinal) were removed, rinsed with physiological saline, weighed, and stored at −70 °C until analysis. The current study protocol was approved by the Ethics Committee of Kyungpook National University for animal studies.

**Table 2:** Composition of experimental diets (%).

Component	NC ^a^	HF	HF-NW	HF-NB	HF-GW	HF-GB
Casein	20.00	23.31	20.92	20.22	20.62	20.16
Sucrose	0.30	20.14				
Dextrose	50.00	11.65				
Corn starch	15.00	8.48	3.05	4.76	3.76	5.23
Cellulose	5.00	5.83	5.63	4.61	5.43	4.48
Soybean Oil	5.00	2.91	2.91	2.91	2.91	2.91
Lard		20.69	20.49	20.50	20.29	20.23
Mineral mixture ^b^	3.50	5.24	5.24	5.24	5.24	5.24
Vitamin mixture ^c^	1.00	1.17	1.17	1.17	1.17	1.17
l-Cystine	0.35	0.35	0.35	0.35	0.35	0.35
Choline bitartrate	0.23	0.23	0.23	0.23	0.23	0.23
NW			40.00			
NB				40.00		
GW					40.00	
GB						40.00
Total (%)	100	100	100	100	100	100
Energy (kcal/100 g)	386	466	466	466	466	466

^a^ NC, normal control diet; HF, high fat diet; HF-NW, high fat diet + instant normal white rice; HF-NB, high fat diet + instant normal brown rice; HF-GW, high fat diet + instant giant embryonic white rice; HF-GB, high fat diet + instant giant embryonic brown rice; ^b^ AIN-76 vitamin mixture; ^c^ AIN-76 mineral mixture.

### 2.4. Analysis of the Plasma and Hepatic Lipids

The plasma triglyceride, total cholesterol, and high-density lipoprotein (HDL) cholesterol concentrations were analyzed using a commercial kit (Asan Pharmaceutical, Soeul, Korea). The hepatic lipids were extracted and purified using the method described by Folch *et al.* [11]. The triglyceride and cholesterol concentrations in liver were measured using the same enzymatic kit used in the plasma analysis. The free fatty acid and phospholipid levels were determined using commercial assay kits (Enzychrom, Bio-Assays Systems, Hayward, CA, USA) following the instruction manual.

### 2.5. Measurement of Glutamate Oxaloacetate (GOT) and Glutamate Pyruvate Transaminase (GPT) Levels

The GOT and GPT levels were measured using a commercial kit (Sigma Chemical Co., St. Louis, MO, USA) based on the method of Reitman and Frankel [12].

### 2.6. Determination of the Lipid-Regulating Enzyme and β-Oxidation Activities

The liver was homogenized in 3–5 mL of 0.1 M triethanolamine, 0.02 M EDTA, and 0.002 M dithiothreitol (DTT) per gram tissue and centrifuged at 1000× *g* at 4 °C for 15 min [13]. The pellet was removed and the supernatant was centrifuged at 10,000× *g* at 4 °C for 15 min. After centrifugation, the pellets were resuspended in the same buffer used in homogenization and analyzed for carnitine palmitoyl transferase (CPT) and β-oxidation activities, and the supernatant was further centrifuged at 105,000× *g* at 4 °C for 1 h. The resulting supernatant was analyzed for glucose-6-phosphate dehydrogenase (G6PD) activity, while the pellets were resuspended in the same buffer containing triethanolamine, EDTA, and DTT and analyzed for fatty acid synthase (FAS) and malic enzyme (ME) activities. The protein content was measured using Bradford protein assay [14].

The FAS activity was determined using the spectrophotometric method described by Gibson and Hubbard [15]. The assay mixture contained 125 mM potassium phosphate buffer (pH 7.0), 10 mM EDTA, 10 mM β-mercaptoethanol, 33 μM acetyl-CoA, 100 μM malonyl-CoA, and 100 μM NADPH. The mixture was added with malonyl CoA and the change in absorbance at 340 nm at 30 °C was recorded.

The ME activity was analyzed following the method of Ochoa [16]. The reaction mixture contained 0.4 M triethanolamine (pH 7.4), 30 mM malic acid, 0.12 M MgCl_2_, and 3.4 mM NADP. The absorbance was measured at 340 nm at 27 °C.

The G6PD activity was measured based on the reduction of 6 mM NADP^+^ by G6PD in the presence of glucose-6-phosphate [17]. The enzyme activity was determined by monitoring the increase in absorption of the reaction mixture at 340 nm at 37 °C. The activities of FAS, ME, and G6PD were expressed as nmol or µmol reduced NADPH/min/mg protein.

The CPT activity was measured according to the method of Bieber [18]. The assay mixture contained 116 mM Tris-HCl (pH 8.0), 1.1 mM EDTA, 2.50 mM l-carnitine, 0.5 mM 5,5-dithiobis-2-nitrobenzoic acid, 75 mM palmitoyl-CoA, 0.2% Triton X-100. The reaction was initiated by the addition of 50 μL cytosol and incubated at 25 °C for 2 min. The change in absorbance at 412 nm was measured and the activity was expressed as nmoL or µmol CoASH or oxidized CoA/min/mg protein.

The β-oxidation activity was determined from the final product of NADH by palmitoyl substrates [19]. The assay mixture contained 50 mM Tris-HCl (pH 8.0) 20 mM NAD+, 0.33 M DTT, 1.5% BSA (1.5 g/100 mL), 2% Triton X-100 (2 g/100 mL), 10 mM CoA, 1 mM FAD, 100 mM KCN and 5 mM palmitoyl-CoA. The reaction was initiated by the addition of 10 μL cytosol and incubated at 37 °C for 5 min. The change in absorbance at 340 nm was measured.

### 2.7. Determination of Plasma Adipokine Concentrations

The concentrations of resistin, adiponectin, and tumor necrosis factor (TNF)-α were determined using enzyme-linked immunosorbent assay kits (Shibayagi Co., Gunma, Japan). The leptin level was measured using an enzyme immunoassay kit (Spi-Bio, Montigny le Bretonneux, France).

### 2.8. Statistical Analysis

All data are presented as the mean ± SE. The data was evaluated by one-way ANOVA using a Statistical Package for Social Sciences software program (SPSS Inc., Chicago, IL, USA) and the differences between the means were assessed using Tukey’s test. Statistical significance was considered at *p* < 0.05.

## 3. Results

### 3.1. Body Weight Gain and Organ Weights

Prior to feeding the mice with experimental diets, the body weights were similar across all groups (Table 3). However, at the end of the experimental period, the HF mice exhibited a marked increase in the body weight relative to the NC group (*p* < 0.05). On the other hand, diet supplementation of instant rice significantly suppressed this high fat-induced body weight gain in mice. The feed intake was significantly higher in HF group compared with the other animal groups. Accordingly, the feed efficiency ratio (FER) was highest in HF mice. The energy intake was also highest in HF group and lowest in NC mice. The weights of liver, heart, and kidney significantly increased in HF group. However, addition of instant rice in the diet suppressed this increase in the organ weights. High fat feeding resulted in a substantial increase in the weights of epididymal, perirenal, and inguinal adipose tissues in mice. However, all instant rice-fed groups exhibited significantly lower body fat weight than the HF group (*p* < 0.05). In particular, the mice fed with instant giant embryonic brown rice showed the lowest amount of adipose tissues.

**Table 3:** Body weight gain and weights of organs and adipose tissues in mice fed with high fat diet supplemented with instant cooked rice.

Parameter	NC	HF	HF-NW	HF-NB	HF-GW	HF-GB
Initial weight (g)	13.64 ± 0.56 ^a^	13.48 ± 0.24 ^a^	13.72 ± 0.42 ^a^	13.99 ± 1.16 ^a^	13.79 ± 0.94 ^a^	13.95 ± 0.38 ^a^
Final weight (g)	27.89 ± 0.60 ^b^	34.86 ± 0.63 ^c^	26.11 ± 0.59 ^a^	28.26 ± 1.16 ^b^	28.18 ± 0.91 ^b^	28.30 ± 0.36 ^b^
Weight gain (g/day)	0.23 ± 0.02 ^b^	0.34 ± 0.01 ^c^	0.20 ± 0.01 ^a^	0.23 ± 0.01 ^b^	0.23 ± 0.01 ^b^	0.23 ± 0.01 ^b^
Feed intake (g/day)	3.56 ± 0.25 ^a^	3.72 ± 0.87 ^b^	3.51 ± 0.68 ^a^	3.50 ± 0.37 ^a^	3.49 ± 0.35 ^a^	3.52 ± 0.49 ^a^
Energy intake (kcal/day)	13.46 ± 0.03 ^a^	18.17 ± 0.02 ^c^	16.30 ± 0.03 ^b^	16.28 ± 0.02 ^b^	16.28 ± 0.01 ^b^	16.31 ± 0.01 ^b^
FER	0.07 ± 0.00 ^c^	0.10 ± 0.00 ^d^	0.06 ± 0.00 ^b^	0.07 ± 0.00 ^c^	0.07 ± 0.00 ^c^	0.05 ± 0.00 ^a^
Organ weight (g)						
Liver	4.25 ± 0.17 ^b^	4.85 ± 0.04 ^c^	4.15 ± 0.07 ^a,b^	4.08 ± 0.06 ^a,b^	4.00 ± 0.57 ^a^	4.00 ± 0.12 ^a^
Heart	0.47 ± 0.02 ^c^	0.97 ± 0.02 ^d^	0.44 ± 0.01 ^a,b^	0.46 ± 0.01 ^b,c^	0.43 ± 0.02 ^a^	0.42 ± 0.01 ^a^
Kidney	1.29 ± 0.03 ^a^	1.80 ± 0.02 ^b^	1.24 ± 0.02 ^a^	1.28 ± 0.02 ^a^	1.24 ± 0.02 ^a^	1.28 ± 0.04 ^a^
White adipose tissue weight (g)						
Epididymal	3.20 ± 0.15 ^b^	6.07 ± 0.12 ^d^	4.16 ± 0.15 ^c^	3.07 ± 0.07 ^b^	3.24 ± 0.11 ^b^	2.79 ± 0.20 ^a^
Perirenal	1.29 ± 0.14 ^a^	2.79 ± 0.31 ^d^	2.14 ± 0.04 ^c^	1.51 ± 0.04 ^a^	1.84 ± 0.11 ^b^	1.24 ± 0.06 ^a^
Inguinal	0.95 ± 0.03 ^a,b^	2.03 ± 0.10 ^d^	1.56 ± 0.13 ^c^	1.06 ± 0.04 ^a,b^	1.34 ± 0.12 ^b,c^	0.85 ± 0.06 ^a^

^a–d^ Values are means ± SE (*n* = 8). Means in the same row not sharing a common superscript are significantly different at *p* < 0.05; NC, normal control diet; HF, high fat diet; HF-NW, high fat diet + instant normal white rice; HF-NB, high fat diet + instant normal brown rice; HF-GW, high fat diet + instant giant embryonic white rice; HF-GB, high fat diet + instant giant embryonic brown rice; FER, food efficiency ratio = body weight gain/feed intake.

### 3.2. Plasma and Hepatic Lipid Profile

A marked increase in the plasma and hepatic triglyceride and total cholesterol concentrations were found in mice fed with high fat diet alone (Table 4). On the other hand, diet supplementation of instant rice, particularly giant embryonic brown rice, considerably decreased the lipid levels (*p* < 0.05). Moreover, the HDL-cholesterol concentration significantly increased in all instant rice-fed groups relative to the NC and HF groups. The HF-GB mice showed the highest HDL-cholesterol level among the animal groups. The non-HDL-cholesterol concentration substantially increased in HF mice relative to the NC group, but markedly decreased in rice-fed groups (*p* < 0.05), with the HF-GB mice having the lowest amount. Accordingly, the HDL-cholesterol/total cholesterol ratio (HTR) and atherogenic index (AI) were highest and lowest, respectively, in HF-GB group. The HF mice exhibited a significant increase in the free fatty acid content relative to the NC group. However, the free fatty acid level markedly decreased in all instant rice-fed groups, with the HF-GB group having the lowest content. The phospholipid level was highest in HF-GB and HF-GW groups. The GOT and GPT levels significantly increased with high fat feeding, but considerably decreased with the addition of instant rice (*p* < 0.05), particularly the giant embryonic brown rice, in the high fat diet.

**Table 4:** Plasma lipid profile and GOT and GPT levels in mice fed with high fat diet supplemented with instant cooked rice.

Parameter	NC	HF	HF-NW	HF-NB	HF-GW	HF-GB
*Plasma*						
Triglyceride (mg/dL)	135.74 ± 2.86 ^a^	180.14 ± 1.11 ^b^	132.33 ± 4.30 ^a^	144.52 ± 5.41 ^a,b^	149.27 ± 5.59 ^a,b^	131.62 ± 1.02 ^a^
Total cholesterol (mg/dL)	157.40 ± 5.69 ^c^	192.80 ± 7.28 ^d^	153.38 ± 3.50 ^b,c^	150.37 ± 3.85 ^b,c^	146.24 ± 5.23 ^a,b^	140.06 ± 2.91 ^a^
HDL-cholesterol (mg/dL)	73.60 ± 4.21 ^a^	70.44 ± 0.64 ^a^	85.29 ± 1.12 ^b^	114.50 ± 1.46 ^d^	110.43 ± 1.39 ^c^	123.88 ± 1.19 ^e^
Non-HDL-cholesterol (mg/dL)	83.10 ± 2.01 ^d^	122.65 ± 1.02 ^e^	68.21 ± 0.99 ^c^	35.27 ± 1.54 ^b^	35.47 ± 1.03 ^b^	16.47 ± 1.00 ^a^
HTR (%)	48.14 ± 2.26 ^b^	34.07 ± 1.87 ^a^	56.48 ± 1.49 ^c^	78.74 ± 2.70 ^d^	78.49 ± 3.36 ^d^	88.98 ± 3.06 ^e^
AI	1.14 ± 0.15 ^d^	1.73 ± 0.11 ^e^	0.79 ± 0.03 ^c^	0.31 ± 0.04 ^b^	0.32 ± 0.04 ^b^	0.13 ± 0.03 ^a^
Free fatty acid (mmol/L)	1.28 ± 0.03 ^b^	2.75 ± 0.12 ^d^	1.76 ± 0.05 ^c^	1.22 ± 0.28 ^a,b^	1.23 ± 0.21 ^a,b^	1.15 ± 0.02 ^a^
Phospholipid (mmol/L)	1.02 ± 0.01 ^b^	0.99 ± 0.01 ^a^	1.04 ± 0.01 ^b^	1.04 ± 0.02 ^b^	1.12 ± 0.01 ^c^	1.14 ± 0.01 ^c^
GOT (karman/mL)	24.61 ± 2.20 ^a b^	47.06 ± 2.28 ^d^	33.01 ± 2.07 ^c^	27.51 ± 1.89 ^b^	27.19 ± 1.07 ^b^	22.42 ± 1.29 ^a^
GPT (karman/mL)	25.87 ± 2.59 ^c^	42.25 ± 2.09 ^d^	25.63 ± 1.66 ^c^	19.55 ± 1.57 ^b^	21.83 ± 2.42 ^b,c^	14.83 ± 2.54 ^a^
*Liver*						
Triglyceride (mg/g)	75.25 ± 1.47 ^a^	89.98 ± 2.47 ^c^	86.24 ± 1.57 ^c^	82.36 ± 2.01 ^b^	81.87 ± 1.88 ^b^	79.01 ± 1.85 ^b^
Total cholesterol (mg/g)	3.06 ± 0.05 ^a^	4.21 ± 0.04 ^e^	3.78 ± 0.07 ^d^	3.26 ± 0.04 ^c^	3.27 ± 0.06 ^c^	3.18 ± 0.04 ^b^

^a–e^ Values are means ± SE (*n* = 8). Means in the same row not sharing a common superscript are significantly different at *p* < 0.05; NC, normal control diet; HF, high fat diet; HF-NW, high fat diet + instant normal white rice; HF-NB, high fat diet + instant normal brown rice; HF-GW, high fat diet + instant giant embryonic white rice; HF-GB, high fat diet + instant giant embryonic brown rice; HTR = (HDL-cholesterol/total cholesterol) × 100; AI, atherogenic index = (total cholesterol − HDL-cholesterol)/HDL-cholesterol; GOT, glutamate oxaloacetate; GPT, glutamate pyruvate.

### 3.3. Lipid-Regulating Enzyme and β-Oxidation Activities

The activities of FAS, ME, and hepatic G6PD enzymes significantly increased, while those of the hepatic CPT and β-oxidation decreased, in HF mice relative to the control group (*p* < 0.05) (Table 5). The animals fed with instant rice, on the other hand, showed markedly lower FAS, ME and G6PD activities and higher hepatic CPT and β-oxidation activities compared with the high fat-fed mice. Among the instant rice-fed groups, the HF-GB group exhibited the lowest activities of FAS, ME and G6PD enzymes. It also showed the highest CPT and β-oxidation activities.

**Table 5:** Lipid-regulating enzyme and β-oxidation activities in mice fed with high fat diet supplemented with instant cooked rice.

Parameter	NC	HF	HF-NW	HF-NB	HF-GW	HF-GB
*Hepatic enzyme activity (nmoL/min/mg protein)*						
FAS	7.06 ± 0.62 ^a^	14.38 ± 0.55 ^c^	8.86 ± 0.95 ^b^	8.25 ± 0.87 ^a,b^	8.04 ± 0.45 ^a,b^	7.17 ± 1.23 ^a^
ME	21.05 ± 0.91 ^c^	26.80 ± 1.51 ^d^	18.02 ± 0.52 ^b^	14.58 ± 0.79 ^a^	14.53 ± 0.73 ^a^	13.84 ± 0.74 ^a^
G6PD	2.77 ± 0.25 ^d^	3.09 ± 0.78 ^f^	2.88 ± 0.46 ^e^	2.02 ± 0.74 ^b^	2.16 ± 0.68 ^c^	1.74 ± 0.55 ^a^
CPT	16.60 ± 1.56 ^b,c^	11.35 ± 0.69 ^a^	15.05 ± 0.73 ^b^	17.28 ± 0.43 ^c^	17.88 ± 0.35 ^c^	20.85 ± 0.38 ^d^
β-oxidation	0.22 ± 0.03 ^b^	0.13 ± 0.02 ^a^	0.24 ± 0.02 ^b^	0.61 ± 0.03 ^d^	0.41 ± 0.04 ^c^	0.80 ± 0.01 ^e^
*Adipocyte enzyme activity (µmoL/min/mg protein)*						
FAS	23.83 ± 3.45 ^b^	46.46 ± 3.00 ^c^	27.03 ± 0.82 ^b^	25.27 ± 3.35 ^b^	22.88 ± 1.78 ^b^	14.68 ± 2.09 ^a^
ME	201.57 ± 2.05 ^c^	258.93 ± 6.17 ^d^	203.82 ± 1.69 ^c^	175.48 ± 1.08 ^b^	170.20 ± 0.84 ^b^	163.08 ± 1.87 ^a^
CPT	302.47 ± 0.89 ^a^	286.75 ± 2.38 ^a^	307.29 ± 2.13 ^a^	382.26 ± 10.05 ^b^	364.59 ± 26.57 ^b^	427.59 ± 1.82 ^c^
β-oxidation	1.07 ± 0.28 ^a,b^	0.66 ± 0.24 ^a^	1.12 ± 0.23 ^a,b^	1.14 ± 0.07 ^a,b^	1.27 ± 0.22 ^b^	1.46 ± 0.52 ^b^

^a–f^ Values are means ± SE (*n* = 8). Means in the same row not sharing a common superscript are significantly different at *p* < 0.05; NC, normal control diet; HF, high fat diet; HF-NW, high fat diet + instant normal white rice; HF-NB, high fat diet + instant normal brown rice; HF-GW, high fat diet + instant giant embryonic white rice; HF-GB, high fat diet + instant giant embryonic brown rice; FAS, fatty acid synthase; ME, malic enzyme; G6PD, glucose-6-phosphate dehydrogenase; CPT, carnitine palmitoyl transferase.

### 3.4. Plasma Adipokine Concentrations

The concentrations of leptin, resistin, and TNF-α markedly increased, while that of the adiponectin decreased, in HF mice compared with the NC group (*p* < 0.05) (Table 6). On the other hand, dietary feeding of instant rice counteracted the elevation in the leptin, resistin and TNF-α concentrations and the reduction in adiponectin level. The HF-GW and HF-GB showed the lowest resistin and TNF-α concentrations among the animal groups. In addition, the leptin and adiponectin levels were lowest and highest in HF-GB mice, respectively.

**Table 6:** Plasma adipokine concentrations in mice fed with high fat diet supplemented with instant cooked rice.

Group	Leptin (ng/mL)	Resistin (ng/mL)	Adiponectin (μg/mL)	TNF-α (ng/mL)
NC	1.48 ± 0.26 ^c^	19.26 ± 1.71 ^a,b^	8.24 ± 0.16 ^b^	1.11 ± 0.04 ^a,b^
HF	1.96 ± 0.04 ^e^	28.71 ± 1.31 ^c^	8.04 ± 0.36 ^a^	1.32 ± 0.02 ^c^
HF-NW	1.53 ± 0.35 ^c^	21.48 ± 1.18 ^b^	8.23 ± 0.09 ^b^	1.17 ± 0.05 ^b^
HF-NB	1.33 ± 0.01 ^b^	19.16 ± 0.95 ^a,b^	8.22 ± 0.04 ^b^	1.17 ± 0.04 ^b^
HF-GW	1.36 ± 0.03 ^b^	18.95 ± 1.38 ^a^	8.37 ± 0.20 ^c^	1.05 ± 0.04 ^a^
HF-GB	1.18 ± 0.04 ^a^	18.42 ± 0.63 ^a^	8.77 ± 0.20 ^d^	1.04 ± 0.19 ^a^

^a–e^ Values are means ± SE (*n* = 8). Means in the same column not sharing a common superscript are significantly different at *p* < 0.05; NC, normal control diet; HF, high fat diet; HF-NW, high fat diet + instant normal white rice; HF-NB, high fat diet + instant normal brown rice; HF-GW, high fat diet + instant giant embryonic white rice; HF-GB, high fat diet + instant giant embryonic brown rice.

## 4. Discussion

Instant foods, including instant rice products, are becoming widely popular due to the modern and busy lifestyles of consumers. With the rising trend in the global incidence of various metabolic diseases, particularly obesity and its related health problems, there is a growing public interest in functional foods that could lower the amount of body fat and improve the lipid profile. In the present study, the comparative effects of dietary feeding of instant cooked rice made from normal white rice, normal brown rice, giant embryonic white rice, and giant embryonic brown rice on the body weight and lipid metabolism in mice under high fat diet conditions were investigated. The results showed that instant cooked rice could suppress body weight gain, decrease body fat, reduce the amounts of plasma and hepatic triglyceride, total cholesterol, and non-HDL-cholesterol concentrations, and increase the HDL-cholesterol level in high fat-fed mice. Among the instant rice samples, the giant embryonic brown rice and normal white rice were the most and least effective, respectively, in reducing the amount of body fat and improving the lipid profile in mice. While the HF-NW mice showed the lowest final body weight and body weight gain among the animal groups, their adipose tissue weights were relatively high, suggesting that the animals might have normal weight obesity. Normal weight obesity is associated with having a normal body weight but high body fat and presents a high risk for developing metabolic syndrome [20,21]. The HF-GB mice also showed significantly lower AI, which measures coronary heart disease risk, than the other groups, indicating that the instant cooked giant embryonic brown rice could reduce the risk of atherosclerosis. Moreover, the levels of plasma GOT and GPT enzymes, which are specific markers of liver damage, were lowest in HF-GB mice, suggesting decreased hepatic oxidative stress in these animals under high fat diet conditions.

Although the instant giant embryonic white and brown rice showed higher body fat-lowering effect and hypolipidemic activity than the normal rice, it is interesting to note that the mice fed with normal white and brown rice exhibited generally similar lipid profile to those fed with control diet. It has been previously shown that cooked ordinary rice could prevent hyperlipidemia in high fat-fed hamsters through regulation of hepatic genes involved in lipid metabolism [22]. Recent studies also revealed that brown rice could prevent obesity and diabetes in humans [23]. Lee *et al.* reported that compared with the conventional brown rice, the giant embryonic brown rice significantly increased the HDL-cholesterol concentrations and reduced the atherosclerosis cardiovascular disease risk in rats fed with high cholesterol diet [24]. It was also reported that diet supplementation of giant embryonic rice markedly decreased the body weight, increased the antioxidative enzyme activities, and reduced the hepatic oxidative damages in diabetic rats [25]. Giant-embryo mutant rice has improved nutritional quality, containing higher amounts of protein, oil, minerals, amino acids, vitamins, GABA, and γ-oryzanol than the normal embryo rice [2,5]. The GABA, in particular, is believed to be one of the active components responsible for the hypocholesterolemic action of germinated giant embryonic rice [24]. GABA is widely known for its health-promoting properties, such as antihypertensive, anticancer, hypolipidemic, and hepatoprotective effects [2,24,26]. Likewise, γ-oryzanol has been shown to possess hypolipidemic, hepatoprotective, and antioxidant properties [27,28,29]. In the present study, the instant cooked giant embryonic white and brown rice contained higher amounts of GABA and γ-oryzanol than their respective normal embryo counterparts, which could be partly responsible for their greater body fat-lowering effect and hypolipidemic action. Moreover, the normal brown rice, which exhibited generally similar functional activity with the white giant embryonic rice, also contained a relatively high amount of GABA and γ-oryzanol. Although the giant embryonic white rice could inhibit hyperlipidemia and body fat accumulation in high fat-fed mice, the inclusion of rice bran further enhanced its physiological activity. Results of the present study showed that instant cooked rice made from giant embryonic brown rice has strong hypolipidemic and body fat-lowering effects and may be useful as a functional food for the prevention and management of high fat diet-induced hyperlipidemia and obesity. Further studies are needed, however, on the bioactive components of this instant giant embryonic rice to better understand its therapeutic potential.

The suppression of lipogenesis through regulation of the lipogenic enzyme activities could be partly responsible for the reduction in the plasma triglyceride, total cholesterol, and fatty acid levels in instant rice-fed groups. The enzymes FAS, ME, G6PD, and CPT are involved in the biosynthesis of cholesterol and fatty acids, and a decrease in the activities of FAS, ME, and G6PD could limit the availability of fatty acids necessary for the triglyceride synthesis [30]. An elevation in the CPT activity, on the other hand, could lead to increased fatty acid oxidation, thereby decreasing the accumulation of triglycerides [31]. Hence, the reduced activities of FAS, ME, G6PD enzymes and increased CPT enzyme activity in instant rice-fed mice might have resulted in the decreased levels of triglyceride, total cholesterol, and fatty acids observed in these animals. Furthermore, the reduction in the amount of body fat in instant rice-fed groups, particularly the HF-GB mice, was probably associated with the decreased enzyme activities of FAS, ME, and G6PD. Studies have shown that enhanced gene expression of FAS was related to visceral fat accumulation in human subjects, while the down regulation of ME and G6PD gene expressions was associated with reduced fat accumulation [32,33].

The hypolipidemic effect of the instant rice samples could also be partly due to the marked reduction in the concentrations of the plasma adipokines leptin, resistin, and TNF-α and elevation in the plasma adiponectin level. These adipokines are protein hormones secreted by the adipose tissues that regulate the lipid and glucose metabolisms [34]. The expression and secretion of leptin, resistin, and TNF-α have been associated with the progression of obesity, while the adiponectin has been shown to be inversely related with obesity and has anti-inflammatory and anti-atherosclerotic properties [35,36]. Hence, the decreased levels of leptin, resistin, and TNF-α and elevated adiponectin concentration found in instant rice-fed mice, particularly the HF-GB mice, relative to the HF group indicate that the production and release of these adipokines may have been influenced by the reduced amount of body fat, resulting in the decreased concentrations of plasma triglyceride and total cholesterol in these animal groups.

## 5. Conclusions

In conclusion, dietary feeding of instant cooked rice made from a giant embryo mutant could control body weight gain, reduce the amount of body fat, and improve lipid profile via inhibition of lipogenesis and regulation of adipokine production in mice under high fat diet conditions. Results indicate that the giant embryonic brown rice has greater hypolipidemic and body fat-lowering effects compared with the giant embryonic white or normal brown rice. This study provides evidence of the physiological activity of instant cooked giant embryonic rice, suggesting that this rice mutant may be beneficial as a functional biomaterial for the development of instant rice with strong anti-obesity effects and hypolipidemic action.
